# Electrochemical DNA biosensor based on the BDD nanograss array electrode

**DOI:** 10.1186/1752-153X-7-65

**Published:** 2013-04-10

**Authors:** Huali Jin, Min Wei, Jinshui Wang

**Affiliations:** 1 College of Food Science and Technology, Henan University of Technology, Zhengzhou, 450001, P. R. China; 2 College of Biological Engineering, Henan University of Technology, Zhengzhou, 450001, P. R. China

**Keywords:** DNA biosensor, AuNPs/CA/nBDD, AuNPs/CA/fBDD

## Abstract

**Background:**

The development of DNA biosensor has attracted considerable attention due to their potential applications, including gene analysis, clinical diagnostics, forensic study and more medical applications. Using electroactive daunomycin as an indicator, the hybridization detection was measured by differential pulse voltammetry in this study.

**Results:**

Electrochemical DNA biosensor was developed based on the BDD film electrode (fBDD) and BDD nanograss array electrode (nBDD). In comparison with fBDD and AuNPs/CA/fBDD electrode, the lower semicircle diameter of electrochemical impedance spectroscopy obtained on nBDD and AuNPs/CA/nBDD electrode indicated that the presence of nanograss array improved the reactive site, reduced the interfacial resistance, and made the electron transfer easier. Using electroactive daunomycin as an indicator, the hybridization detection was measured by differential pulse voltammetry.

**Conclusions:**

The experimental results demonstrated that the prepared AuNPs/CA/nBDD electrode was suitable for DNA hybridization with favorable performance of faster response, higher sensitivity, lower detection limit and satisfactory selectivity, reproducibility and stability.

## Background

The development of DNA biosensor has attracted considerable attention due to their potential applications, including gene analysis, clinical diagnostics, forensic study and more medical applications [1,2]. Among the various methods for DNA detection, electrochemical techniques offer great advantages with simplicity, rapidness, relatively low cost, and high sensitivity, and are suitable for the development of inexpensive and portable devices [3-5].

The immobilization of DNA onto the electrodes plays an important role in the fabrication of DNA electrochemical biosensors. With the properties of high surface-to-volume ratio, unique catalytic activity, high electron transfer rate, and strong adsorption ability, gold (Au) nanomaterials with different morphology have been introduced onto the electrode surface through the Au-thiol binding [6-10] or direct immobilization [11-15] for the loading of DNA.

On the other hand, with the advantages of wide potential window, low background current, high stability and excellent resistance to electrode fouling, boron-doped diamond (BDD) film (fBDD) electrode has been used widely for electrochemical detection. In order to add advanced functions, BDD nanograss array electrode (nBDD) was prepared in our previous work [16].

In the present work, electrochemical DNA biosensor was developed based on the fBDD and the nBDD electrode. The obtained biosensors were characterized by electrochemical impedance spectroscopy (EIS). Probe DNA with thiol group at 5’ end was covalently linked onto the electrode surface through the Au-thiol binding. Hybridization experiment was conducted by immersing the electrode with probe DNA into the hybridization buffer solution containing its complementary sequence. Using electroactive daunomycin (DNR) as an indicator, the hybridization detection was measured by differential pulse voltammetry (DPV).

## Experimental

### Chemicals and apparatus

The oligonucleotides were chosen according to the literature [15] and purchased from TaKaRa Biotechnology Co., Ltd. (Dalian, China).; their base sequences are as follows:

24-base probe sequence (probe DNA): 5^′^-SH-GAG CGG CGC AAC ATT TCA GGT CGA-3^′^

its fully complementary sequence: 5^′^-TCG ACC TGA AAT GTT GCG CCG CTC-3^′^

a non-complementary sequence: 5^′^-GAG CGG CGC AAC ATT TCA GGT CGA-3^′^

Others sequences as following:

5^′^-TCG ACC TGA AA*C* GTT GCG CCG CTC-3^′^ (one-base mismatch);

5^′^-TCG ACC T*C*A AAT GTT G*A*G CCG CTC-3^′^ (two-base mismatch);

5^′^-TCG *T*CC TGA AA*C* GTT GCG CC*T* CTC-3^′^ (three-base mismatch).

Daunomycin hydrochloride was obtained from Shanghai Institute for Drug Control and used without further purification. 10 × TE buffer (0.1 M Tris–HCl, 0.01 M EDTA, pH 8.0) and wash buffer (PBS, 0.01 M phosphate sodium buffer solution, pH 7.4, 0.1 M NaCl) from Shanghai Your Sun Biological Technology Co., Ltd. were all used as-received. Hybridization buffer (0.75 M NaCl, 0.15 M sodium citrate, pH 7.4) was self-prepared. All other reagents were of analytical grade and Millipore Milli-Q ultrapure water was used throughout.

Electrochemical measurements were performed on a CHI660c electrochemical workstation with a three-electrode electrochemical cell. A Ag/AgCl (saturated KCl) reference electrode and a Pt wire counter electrode were used.

### Preparation of the AuNPs/CA/fBDD and AuNPs/CA/nBDD electrode

**Scheme 1:**
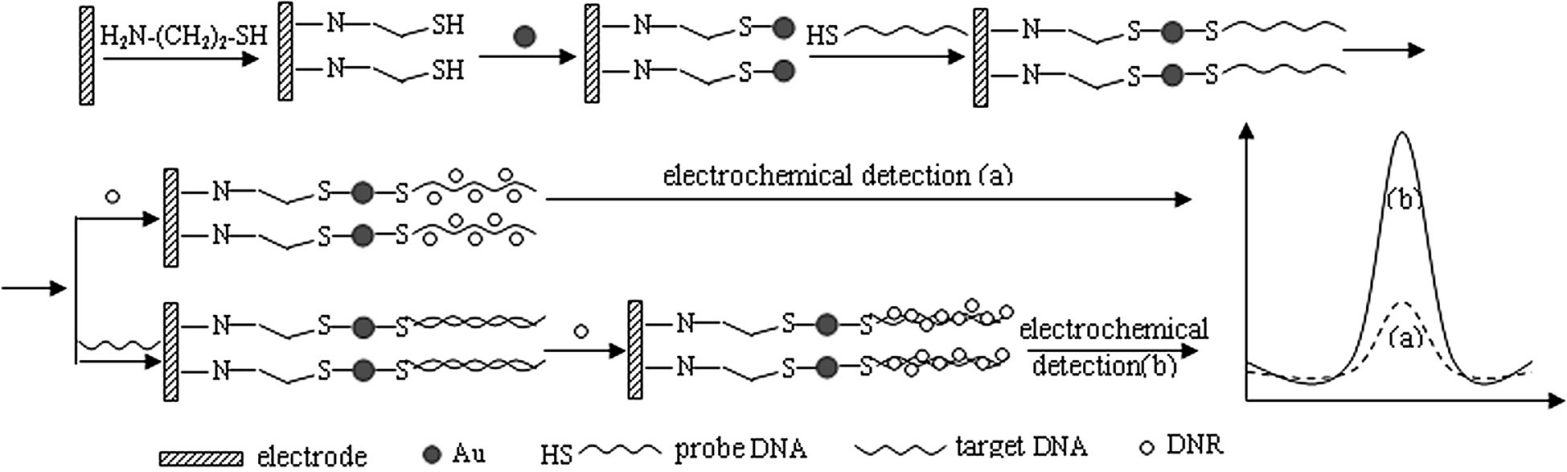
The procedure for preparation of DNA biosensor and detection of DNA hybridization.

The procedure for preparation of the AuNPs/CA/fBDD and AuNPs/CA/nBDD electrode was described in Scheme 1. Firstly, Au nanoparticles (AuNPs) were prepared according to the literature [17]. Cyclic voltammetry (CV) was used to immobilize cysteamine (CA) onto surface of the pretreated fBDD and nBDD electrode at the range from −1.5 V to + 2.4 V in PBS (0.1 M, pH 7.0) containing1.0 × 10^−3^ M CA [18]. Then the CA modified electrodes were rinsed thoroughly with ultrapure water and immersed in AuNPs solution for 1 h. With the strong interaction between Au and the thiol, the AuNPs could be linked to the CA modified fBDD and nBDD.

### Hybridization and electrochemical detection

Scheme 1 outlined the procedure of DNA hybridization and electrochemical detection on the prepared electrodes. The concrete experimental processes were as follows:

Probe DNA with thiol group at 5’ end was covalently linked onto the electrode surface through the Au-thiol binding, and the processes were as follows: The prepared electrode was immersed into a 1.58 × 10^−5^ M probe DNA soultion for 12 h with stirring at room temperature. Then the electrode was washed using wash buffer to remove the unimmobilized probe DNA.

The probe DNA-modified electrode was immersed into hybridization buffer solution containing different concentration of target DNA for 30 min at 37°C. After that, the electrode was soaked in wash buffer for removing the non-hybridized target DNA. Then, the hybridized electrode was immersed into the stirred DNR solution (1.0 × 10^−5^ M) containing PBS (0.1 M, pH 7.4) for 15 min at room temperature. After accumulation of DNR, the electrode was rinsed in PBS (0.1 M, pH 7.4) for 30 s to remove the physically adsorbed molecules [19]. Using the hybridized electrode with accumulated DNR as working electrode, the hybridization detection was measured by DPV in PBS soultion (0.1 M, pH 7.4).

## Results and discussion

### Characterization of the AuNPs/CA/fBDD and AuNPs/CA/nBDD electrode

**Figure 1:**
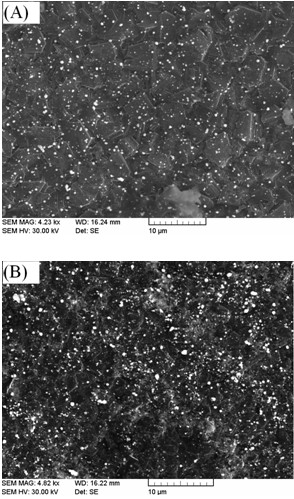
SEM images of AuNPs/CA/fBDD (A) and AuNPs/CA/nBDD (B).

Figure 1 showed the SEM images of AuNPs/CA/fBDD (Figure 1A) and AuNPs/CA/nBDD (Figure 1B) electrode. As shown in Figure 1, AuNPs were successfully immobilized onto SH-terminated fBDD and nBDD surfaces randomly. The surface coverage of AuNPs on electrode surface was estimated by manually counting the AuNPs amount in different analysis areas (1 μm × 1 μm) in SEM images and the average density of nanoparticles was adopted as coverage [20]. The obtained coverage of AuNPs is about 1.31 × 10^8^ particles/cm^2^ on fBDD surface and 5.75 × 10^8^ particles/cm^2^ on nBDD surface, indicating that the presence of nanograss array on the electrode surface could accelerate the immobilization of AuNPs.

**Figure 2:**
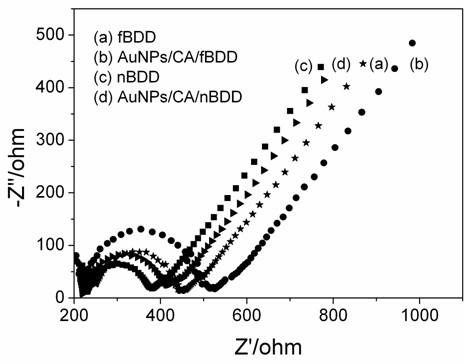
**Nyquist plots of 10 mM [Fe(CN)**_**6**_**]**^**3-/4- **^**in 0.1 M KCl from 10**^**5**^**Hz to 0.01 Hz at an ac amplitude of 10 mV under open-circuit potential conditions, obtained at (a) fBDD, (b) AuNPs/CA/fBDD, (c) nBDD, (d) AuNPs/CA/nBDD.**

Figure 2 showed the results of EIS for 10 mM [Fe(CN)_6_]^3-/4-^ in 0.1 M KCl on the different electrode surface. The obvious EIS change from both Figure 2a to 2b and 2c to 2d demonstrated that CA and AuNPs successfully bound to surface of fBDD and nBDD. The lower semicircle diameters were obtained on the nBDD electrode (Figure 2c) and the AuNPs/CA/nBDD electrode (Figure 2d), compared to that obtained on the fBDD electrode (Figure 2a) and the AuNPs/CA/fBDD electrode (Figure 2b), indicating that the presence of nanograss array on the electrode surface improved the reactive site, reduced the interfacial resistance, and made the electron transfer easier.

### Detection of DNA hybridization on the AuNPs/CA/fBDD and AuNPs/CA/nBDD electrode

**Figure 3:**
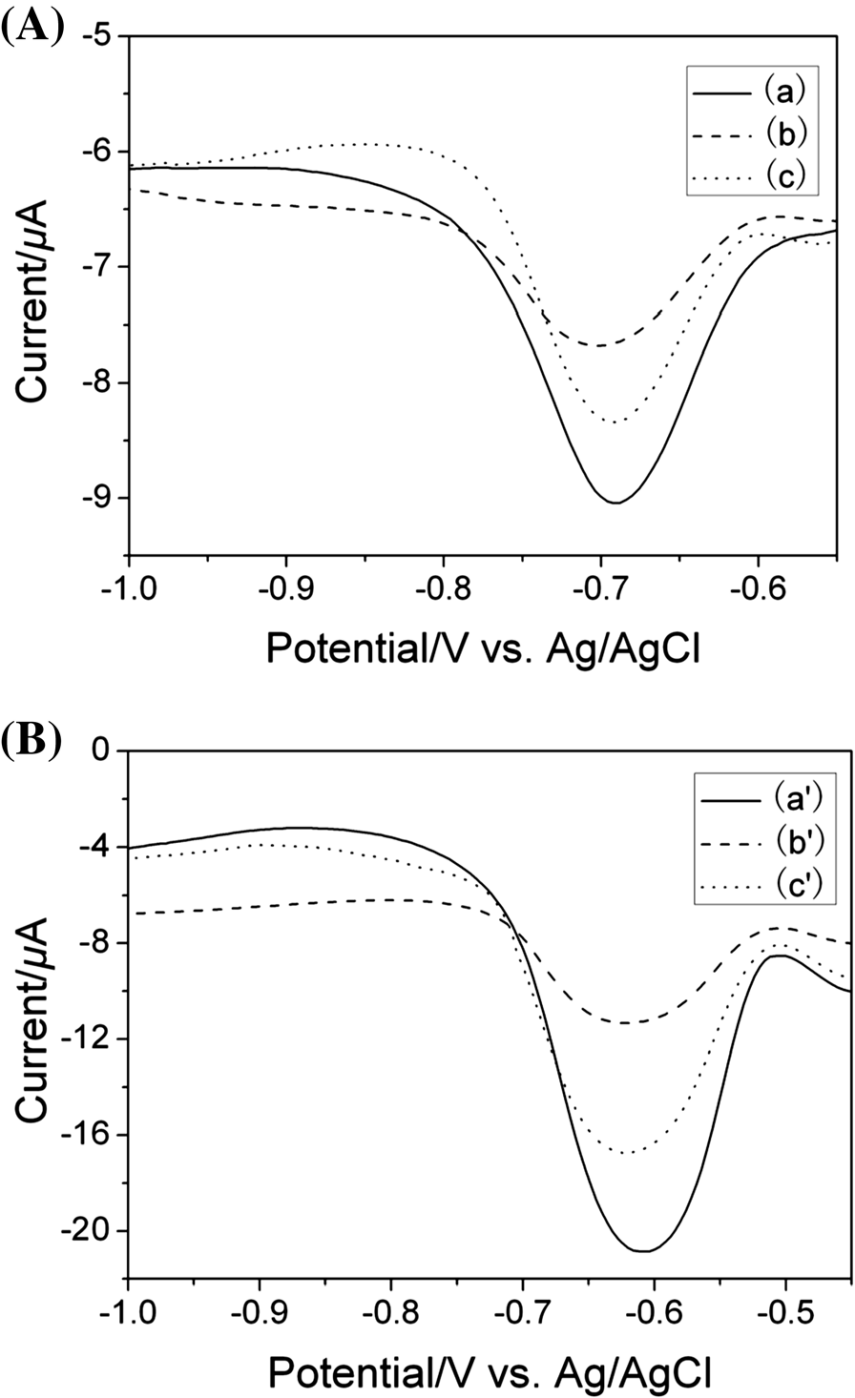
**The different DPV responses obtained on the AuNPs/CA/fBDD electrode (Figure**3**(A)) and the AuNPs/CA/nBDD electrode (Figure**3**(B)). (a)** and (a’), the DPV responses of DNR solution (1.0 × 10^−5^ M) containing PBS (0.1 M, pH 7.4) without ssDNA probe; **(b)** and (b’), the DPV responses of DNR solution (1.0 × 10^−5^ M) containing PBS (0.1 M, pH 7.4) in the presence of ssDNA probe; **(c)** and (c’), the DPV responses of the hybridized electrodes with accumulated DNR in PBS (0.1 M, pH 7.4). target DNA: 1.58 × 10^−7^ M. DPV parameters: Amplitude: 0.05 V; pulse period: 0.2 s; pulse width: 0.05 s.

Figure 3 illustrated the different DPV responses of DNR solution (1.0 × 10^−5^ M) containing PBS (0.1 M, pH 7.4) without ssDNA probe (a and a’), in the presence of ssDNA probe (b and b’), and those of the hybridized electrodes with accumulated DNR in PBS (0.1 M, pH 7.4) (c and c’) on the AuNPs/CA/fBDD electrode (Figure 3(A)) and that on the AuNPs/CA/nBDD electrode (Figure 3(B)). As shown in Figure 3a and 3a’, the two electrodes not containing ssDNA showed the good electrochemical responses in DNR solutions. The reduction peak potential of DNR positively shifted from about −0.69 V on the AuNPs/CA/fBDD electrode to about −0.60 V on the AuNPs/CA/nBDD electrode, and the reduction peak current increased from 2.3 μA to 12.33 μA, i.e., on the AuNPs/CA/nBDD electrode, the reduction peak potential shifted positively by ~0.09 V and the peak current increased by almost 5 times, compared to those on the AuNPs/CA/fBDD electrode. The results demonstrated that the reduction process of DNR was improved greatly on the AuNPs/CA/nBDD electrode, which was ascribed that nanograss array could accelerate the speed of electron transfer and increase the efficient area of electrode.

The immobilization of ssDNA probe onto the electrodes resulted in obvious decreases in DPV responses of DNR (b and b’), which was attributed to the less DNR molecules accessibility to ssDNA probe on the electrode surface [21]. The results also showed that the ssDNA probe had been successfully immobilized on the two electrodes.

After hybridization and DNR intercalation, DPV responses of the hybridized electrodes with accumulated DNR in PBS (0.1 M, pH 7.4) (c and c’) were depicted. As shown in Figure 3c and 3c’, the pronounced increase in the peak current of the DNR located respectively at about −0.69 V and about −0.60 V was observed when hybridized with its complementary sequence. This was ascribed that more DNR could intercalate in the DNA double helix after hybridization, which gave an increased electrochemical response.

In comparison with the AuNPs/CA/fBDD electrode, on the AuNPs/CA/nBDD electrode, the positive shift of peak potential and the obvious increase of all DPV responses demonstrated that the AuNPs/CA/nBDD electrode had advantages with faster response and higher sensitivity and was superior to the AuNPs/CA/fBDD electrode for DNA hybridization.

### Selectivity and calibration curve of DNA hybridization on the AuNPs/CA/nBDD electrode

**Figure 4:**
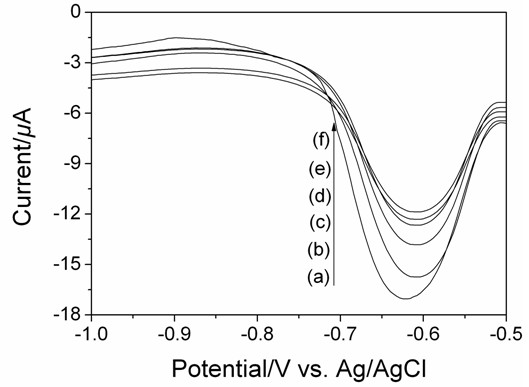
**The DPV response of DNR (1.0 × 10**^**−5**^ **M) as indicator in PBS (0.1 M, pH 7.4) recorded for the AuNPs/CA/nBDD electrode with probe DNA without hybridization (f), hybridized with non-complementary sequence (e), three-base mismatch sequence (d), two-base mismatch sequence (c), one-base mismatch sequence (b), and complementary sequence (a).** Different target DNA concentration: 1.58 × 10^−7^ M; DPV parameters: Amplitude: 0.05 V; pulse period: 0.2 s; pulse width: 0.05 s.

The selectivity of the DNA hybridization on the AuNPs/CA/nBDD electrode with probe DNA was investigated by hybridizing with its complete complementary sequence, different-base mismatch sequence, and non-complementary sequence. As shown in Figure 4, as compared with the DPV response of DNR with probe DNA (Figure 4f), a pronounced increase in the peak current value of the DNR was observed when hybridized with its complementary sequence (Figure 4a). The DPV signal was gradual decreasing with the increase of the mismatch numbers of target DNA (Figure 4b to 4d), and the negligible electrochemical response was obtained when incubating with non-complementary sequence (Figure 4e). The results showed that the AuNPs/CA/nBDD electrode had satisfactory selectivity for DNA hybridization.

**Figure 5:**
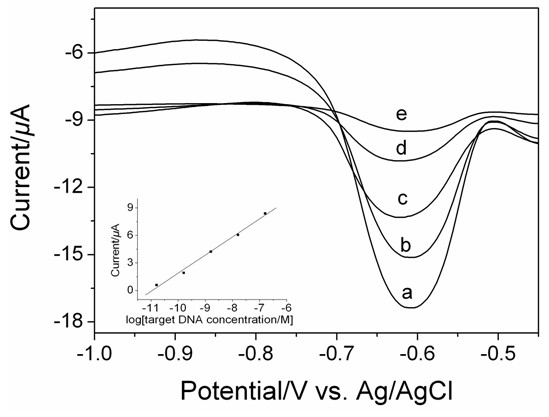
**The DPV responses of DNR as indicator in PBS (0.1 M, pH 7.4) for different target concentrations: (a) 1.58 × 10**^**−7**^ **M, (b) 1.58 × 10**^**−8**^ **M, (c) 1.58 × 10**^**−9**^ **M, (d) 1.58 × 10**^**−10**^ **M, (e) 1.58 × 10**^**−11**^ **M.**

Figure 5 displayed the DPV hybridization responses for increasing target concentrations of complementary sequence from 1.58 × 10^−11^ M to 1.58 × 10^−7^ M. The peak currents of intercalated DNR increased with the increase of the complementary target DNA concentrations and were linear with the logarithmic value of the complementary target DNA concentrations, as shown in the inset of Figure 5. The linear regression equation was *y* = 1.972 log *x +* 21.5996 with a correlation coefficient value of 0.996 (*x* is the concentration of complementary sequence, M; *y* the peak current of DNR, μA). The detection limit of 9.8 × 10^−12^ M was obtained using 3 *s* (*s* is the standard deviation of blank solution, *n* =10), which was lower than the value of previous report for the same target sequence detection [22].

### Reproducibility and stability of the DNA biosensor

A series of 8 independent measurements of 1.58 × 10^−7^ M target DNA were used for estimating the precision, and the relative standard deviation (RSD) was 5.63%. It showed that the proposed DNA biosensor had acceptable reproducibility. The probe DNA modified electrode was first stored in the refrigerator at 4°C over three weeks and then examined via DPV after its hybridization with complementary target DNA. Experiments demonstrated that the DNA biosensor retained about 91% of its initial response.

## Conclusions

An electrochemical DNA biosensor was developed based on the AuNPs/CA/nBDD electrode. Both the lower semicircle diameter of EIS and the favorable results of hybridization detection obtained on the AuNPs/CA/nBDD electrode indicated that the presence of nanograss array could improve the reactive site, increase the efficient area of electrode, reduce the interfacial resistance, and make the electron transfer easier. The prepared AuNPs/CA/nBDD electrode was suitable for DNA hybridization with favorable performance of faster response and higher sensitivity, lower detection limit and satisfactory selectivity, reproducibility and stability.

## Competing interests

The authors declare that there are no any competing interests.

## Authors’ contributions

MW planed and supervised the whole work, and drafted the manuscript. HLJ carried out the experiments and drafted the manuscript. JSW participated in experiments. All authors read and approved the final manuscript.
